# Quantification of preexisting lung ground glass opacities on CT for predicting checkpoint inhibitor pneumonitis in advanced non-small cell lung cancer patients

**DOI:** 10.1186/s12885-024-12008-z

**Published:** 2024-02-26

**Authors:** Xinyue Wang, Jinkun Zhao, Ting Mei, Wenting Liu, Xiuqiong Chen, Jingya Wang, Richeng Jiang, Zhaoxiang Ye, Dingzhi Huang

**Affiliations:** 1 Tianjin Medical University Cancer Institute & Hospital, National Clinical Research Center for Cancer, Tianjin, China; 2 Key Laboratory of Cancer Prevention and Therapy, Tianjin, China; 3 Tianjin’s Clinical Research Center for Cancer, Tianjin, China; 4 Department of Thoracic Oncology, Tianjin Cancer Institute & Hospital, Tianjin Medical University, Huanhuxi Road, Hexi District, 300060 Tianjin, China; 5 Department of Radiology, Tianjin Cancer Institute & Hospital, Tianjin Medical University, Huanhuxi Road, Hexi District, 300060 Tianjin, China

**Keywords:** Non-small cell lung cancer, Immune checkpoint inhibitor, Checkpoint inhibitor pneumonitis, Ground glass opacity, Deep learning

## Abstract

**Background:**

Immune checkpoint inhibitors (ICIs) can lead to life-threatening pneumonitis, and pre-existing interstitial lung abnormalities (ILAs) are a risk factor for checkpoint inhibitor pneumonitis (CIP). However, the subjective assessment of ILA and the lack of standardized methods restrict its clinical utility as a predictive factor. This study aims to identify non-small cell lung cancer (NSCLC) patients at high risk of CIP using quantitative imaging.

**Methods:**

This cohort study involved 206 cases in the training set and 111 cases in the validation set. It included locally advanced or metastatic NSCLC patients who underwent ICI therapy. A deep learning algorithm labeled the interstitial lesions and computed their volume. Two predictive models were developed to predict the probability of grade ≥ 2 CIP or severe CIP (grade ≥ 3). Cox proportional hazard models were employed to analyze predictors of progression-free survival (PFS).

**Results:**

In a training cohort of 206 patients, 21.4% experienced CIP. Two models were developed to predict the probability of CIP based on different predictors. Model 1 utilized age, histology, and preexisting ground glass opacity (GGO) percentage of the whole lung to predict grade ≥ 2 CIP, while Model 2 used histology and GGO percentage in the right lower lung to predict grade ≥ 3 CIP. These models were validated, and their accuracy was assessed. In another exploratory analysis, the presence of GGOs involving more than one lobe on pretreatment CT scans was identified as a risk factor for progression-free survival.

**Conclusions:**

The assessment of GGO volume and distribution on pre-treatment CT scans could assist in monitoring and manage the risk of CIP in NSCLC patients receiving ICI therapy.

**Clinical relevance statement:**

This study’s quantitative imaging and computational analysis can help identify NSCLC patients at high risk of CIP, allowing for better risk management and potentially improved outcomes in those receivingICI treatment.

**Supplementary Information:**

The online version contains supplementary material available at 10.1186/s12885-024-12008-z.

## Background

In advanced non-small cell lung cancer (NSCLC), a growing number of clinical studies have suggested that a lasting response and improvement in long-term survival can be achieved with immune checkpoint inhibitors (ICIs), which target programmed cell death protein-1 (PD-1) or programmed cell death protein ligand-1 (PD-L1) [1–6]. Importantly, ICIs can also cause immune-related adverse events (irAEs), including checkpoint inhibitor pneumonitis (CIP) [7].

CIP is defined as the development of a new infiltrative shadow on chest imaging following the application of ICI therapy, with or without respiratory symptoms. However, identification of a clinical condition, such as a lung infection or tumor progression, will exclude a diagnosis of CIP. According to the data of clinical trials and real-world studies, the incidence of CIP is 3-19% in NSCLC [1–4, 8–12], and it is more prevalent and severe in NSCLC than in other cancers [8, 9]. CIP may result in the discontinuation of ICI therapy permanently, leading to an increased utilization of critical care and risk of mortality for patients [11–14]. The results of previous retrospective studies suggest that advanced age [14], smoking status [15], combination therapy [8], PD-1 inhibitor use (rather than PD-L1 inhibitor use) [10], chest radiation [16, 17], nonadenocarcinoma [11], lower albumin [12], fibrosis score [18] and baseline pulmonary function impairment [19] may be related to the occurrence of CIP.

Despite these risk factors, baseline lung disease, specifically, preexisting interstitial lung disease (ILD), is also independently associated with the development of CIP [14, 20–23]. Caution is warranted because of the high incidence of pneumonitis in patients with ILD. However, the diagnosis of ILD is often challenging due to its nonspecific and insidious presenting symptoms, along with scarce knowledge of ILD among non-ILD experts; therefore, the incidence of ILD among real-world patients with lung cancer is underestimated. Interstitial lung abnormalities (ILAs), which have been defined as areas of increased lung density on lung computed tomography (CT) in individuals with no known ILD [24], are also considered to be a risk factor for CIP [25].

It is important to note that most risk factors mentioned above, such as advanced age, long-term smoking, combined use of targeted therapies, a history of radiation therapy, and baseline lung disease, may increase the likelihood of developing pulmonary interstitial lesions on CT, and in turn, baseline pulmonary function impairment may result from these lesions. It is therefore postulated that preexisting pulmonary interstitial lesions on CT play a crucial role in early detection and management of CIP. In practice, however, pulmonary interstitial lesions are heterogeneous and difficult to evaluate, and it is understandable that radiological examinations are qualitatively interpreted with a certain degree of subjectivity. Thus, quantitative analysis of imaging is likely to become increasingly important in the assessment of interstitial lesions.

Therefore, we hypothesized that the quantified volume and component of interstitial lesions at pretreatment CT would be associated with CIP in patients with NSCLC. We conducted a retrospective analysis to quantify interstitial lesions at pretreatment CT by using a deep learning algorithm and to evaluate their extent, component and distribution as predictors of CIP, as well as to assess the association between pretreatment radiographic findings and progression-free survival (PFS) in patients with advanced NSCLC.

## Materials and methods

### Data sources and participants

**Fig. 1:**
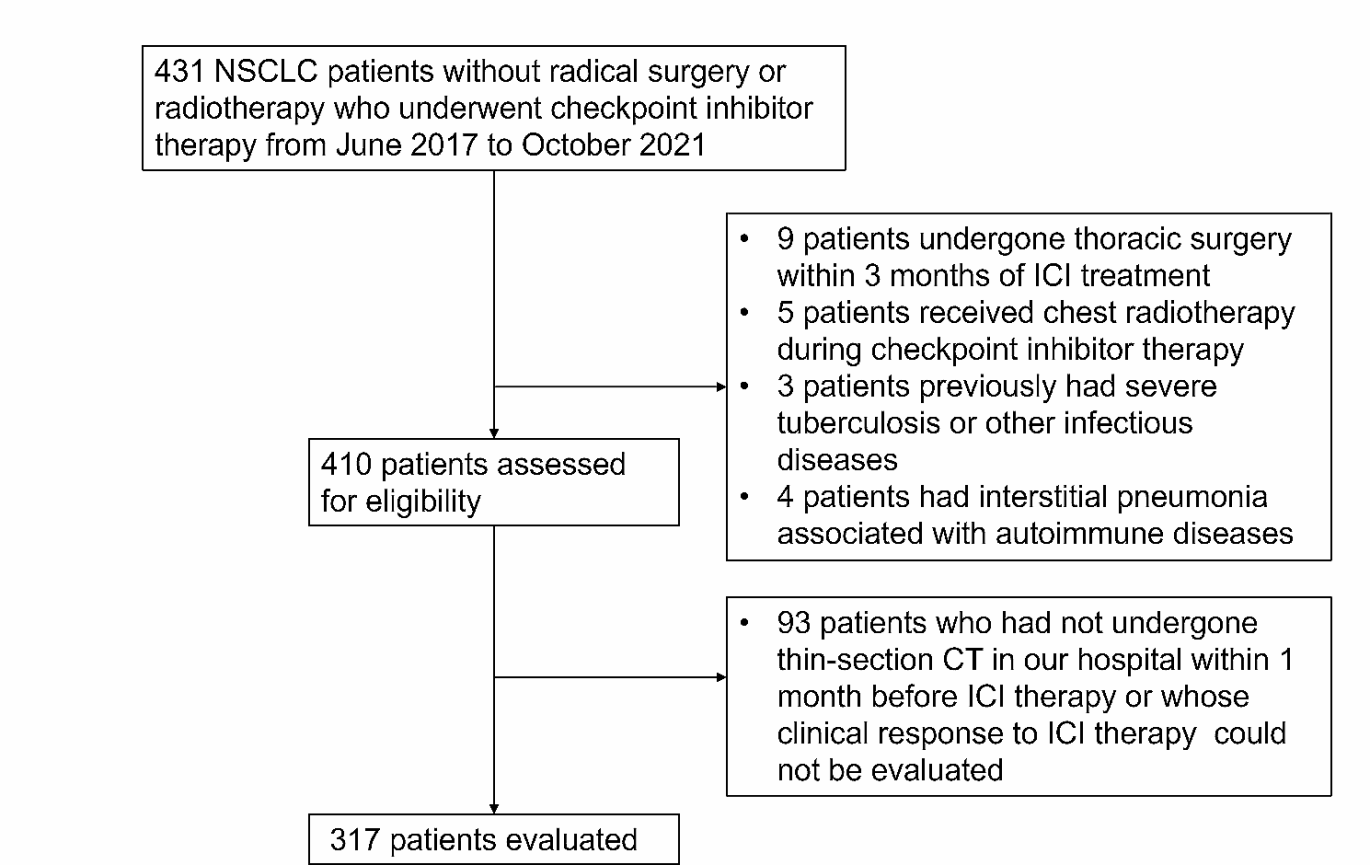
Flowchart of the study. Abbreviation: NSCLC, non-small cell lung cancer. ICI, immune checkpoint inhibitor

This single-center cohort study was approved by the ethics committee of the Tianjin Medical University Cancer Institute & Hospital. To avoid selection bias, consecutive histologically confirmed locally advanced or metastatic NSCLC patients without radical surgery or radiotherapy who underwent checkpoint inhibitor therapy (nivolumab, pembrolizumab, tislelizumab or atezolizumab) from June 2017 to October 2021 in our hospital were potential candidates for this study. We retrospectively reviewed data for 431 patients with complete information about clinicopathological characteristics. Among the 431 patients, we excluded patients who had undergone thoracic surgery within 3 months of ICI treatment (*n* = 9) or had received chest radiotherapy during checkpoint inhibitor therapy (*n* = 5) and patients who had previously had severe tuberculosis or other infectious diseases (*n* = 3) or interstitial pneumonia associated with autoimmune diseases (*n* = 4). We also excluded 93 patients who had not undergone thin-section CT in our hospital within 1 month before ICI therapy or whose clinical response to ICI therapy could not be evaluated. Finally, 206 patients treated before November 2020 were considered the training cohort, and the validation cohort consisted of 111 patients treated between November 2020 and October 2021. Patient characteristics and clinical data before administration of anti-PD-1/PD-L1 antibodies were obtained by using medical records. Tumor responses were evaluated using the Response Evaluation Criteria for Solid Tumors version 1.1. March 15, 2022 was set as the end of the follow-up. The Transparent Reporting of a multivariable prediction model for Individual Prognosis or Diagnosis (TRIPOD) statement was used as the reporting guideline [26]. The flow diagram is shown in Fig. 1.

### CT imaging acquisition and evaluation

Pretreatment thin-section volumetric CT images were performed according to standard noncontrast chest CT protocols in our institution. CT examinations were performed using a Somatom Sensation 64 (Siemens Medical Solutions, Forchheim, Germany) CT scanner or a Discovery CT 750 HD (GE Medical Systems; Milwaukee, USA). The scan tube voltage was 120 kVp with automatic tube current modulation. The scan range included the pulmonary apex level to below the diaphragm. Key imaging parameters such as pitch (0.95/0.984 mm), rotation speed (0.8 /0.6s), kernel (B70f and B30/Stnd and Lung), slice thickness (1.50/1.25 mm), and W/L settings for lung window (window width: 1200, window level: -500) were carefully configured to optimize the image quality and highlight specific tissue contrasts, particularly in the assessment of pulmonary structures.

We investigated the presence of abnormal findings other than lung cancer lesions, including preexisting ILA on CT. ILA was defined as nondependent abnormalities affecting more than 5% of any lung zone (upper, middle, and lower lung zones are demarcated by the levels of the inferior aortic arch and right inferior pulmonary vein) [24]. Two expert chest radiologists (J.Z., with 15 years of experience, and X.C., with 6 years of experience) independently evaluated the CT images in a randomized order without any clinical information. The radiologists evaluated whether ILA existed by using a previously reported scoring system [27], in which a score of 1 indicated no ILA; a score of 2 indicated equivocal ILA; and a score of 3 indicated ILA. A third radiologist (Z.Y., with 25 years of experience) who was blinded to the clinical information provided the majority opinion in cases with discrepant results between the first two radiologists. The radiologists (J.Z. and X.C.) also determined the extent of each fibrosis lesion, and we used the average of the results. The sum of reticulation, honeycombing and traction bronchiectasis was calculated as the fibrosis extent for each examination.

**Fig. 2:**
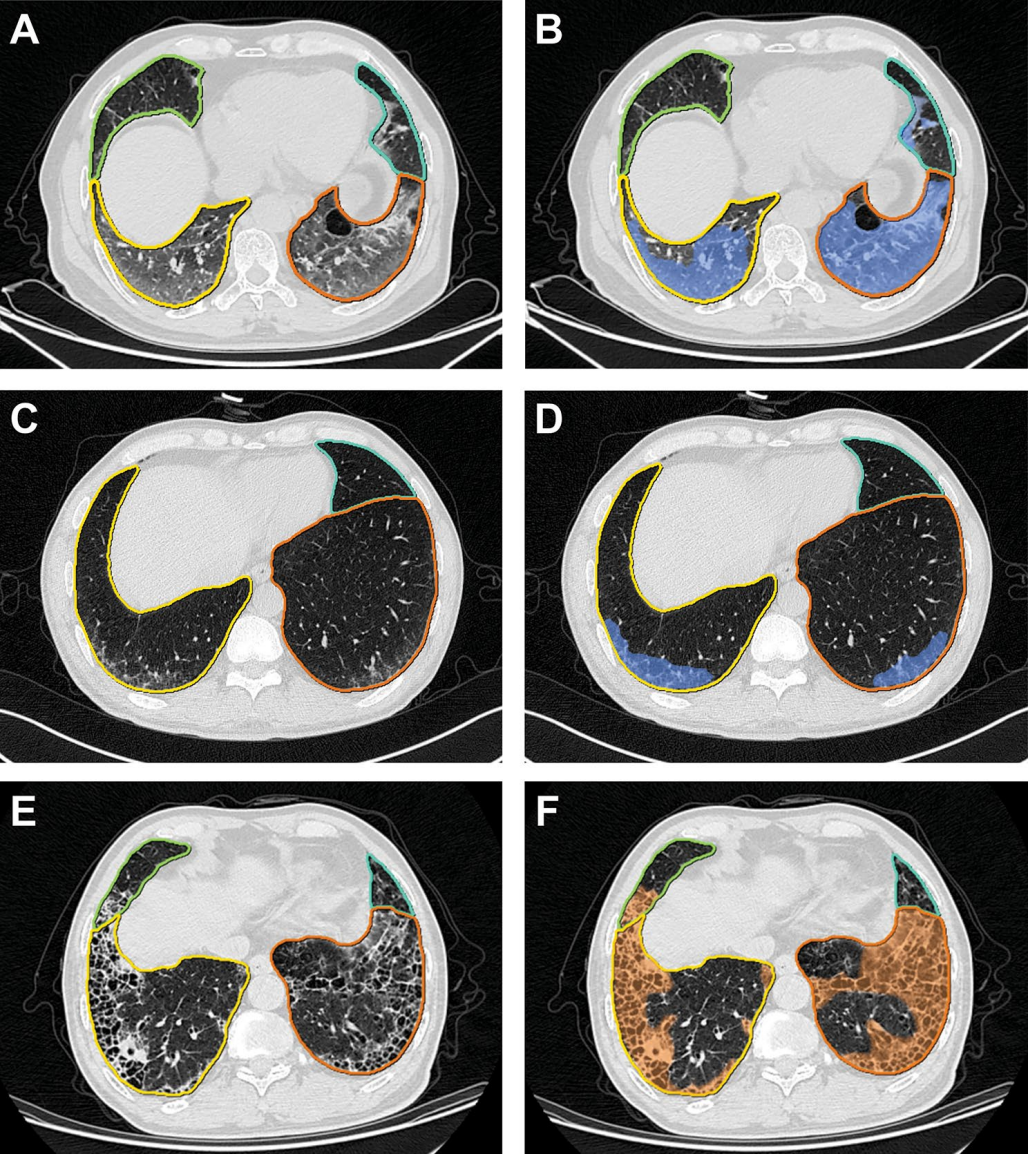
Examples of pulmonary lobe segmentation and opacity segmentation (**A**, **B**), pretreatment CT images of a patient with ground glass opacity, the patient developed grade 3 CIP at 133 days after ICI treatment. (**C**, **D**), pretreatment CT images of a patient with ground glass opacity, the patient developed grade 1 CIP at 118 days after ICI treatment. (**E**, **F**) pretreatment CT images of a patient with fibrosis, the patient did not develop CIP after ICI treatment

Analysis of pretreatment CT images by using the dedicated multitask deep learning algorithm developed for pulmonary pneumonia (Beijing Deepwise & League of PhD Technology Co. Ltd, China) [28]. Further information regarding this approach is reported in the Supplementary Methods (Supplementary Material 1). Fig. 2 shows examples of pulmonary lobe and opacity segmentation. First, we segmented each lung lobe and the bilateral lungs and calculated the volume. Subsequently, fibrosis lesions were labeled by radiologists, while the GGO and consolidation lesions in the corresponding lobes were labeled using the well-trained artificial intelligence (AI) algorithm, and then the volume of each lesion was computed. All opacity pieces extracted by the AI system were checked by a radiologist (J.Z.) to prevent lung cancer lesions from being labeled. The lesion volume percentage in each lobe as well as the number of involved lung lobes were calculated. ILA determined by AI was defined as the sum of GGO and fibrosis extent affecting more than 5% of any lung zone.

### Outcomes

The diagnosis of CIP was first made according to the medical records, and then each case was reviewed to confirm the diagnosis by the oncologist (X.W., R.J.) in consultation with the radiologist (J.Z.) based on previous literature descriptions [15]. By combining clinical data, radiographic data and biologic data, cases with alternative etiologies such as heart failure, infection, and tumor progression were excluded. Each case of pneumonitis was classified according to the American Thoracic Society/European Respiratory Society (ATS/ERS) international multidisciplinary classification of interstitial pneumonia with slight modifications from previous reports on drug-induced ILD with slight modifications from previous reports on drug-induced pneumonitis [17, 29, 30], including cryptogenic organizing pneumonia (COP)-like pattern, GGOs, nonspecific interstitial pneumonia (NSIP)-like pattern, hypersensitive pneumonitis (HP)-like pattern, and others. According to the National Comprehensive Cancer Network (NCCN) guidelines [31], CIP is graded into four levels according to the combination of clinical manifestations and radiological findings. ICI therapy was discontinued in all cases in which a patient was diagnosed with symptomatic CIP (grade ≥ 2 CIP). High-grade CIP (grade ≥ 3 CIP) is more likely to cause treatment interruption or complete withdrawal from ICI treatment and severely decrease pulmonary function, which leads to poor efficacy and quality of life. Corticosteroid treatment was given to all patients with grade ≥ 2 CIP in accordance with current guidelines. A reduction in oxygen requirement, an improvement in exercise capacity, and a decrease in radiographic infiltrates were considered to indicate clinical improvement.

### Data analysis

The detailed methods of data analysis, which encompass data quality and missing data, variable selection, model development, performance evaluation of the model, validation, and statistical analysis, have been provided in Supplementary Methods (Supplementary Material 1).

## Results

### Study population characteristics

The characteristics of the training cohort (*n* = 206) and validation cohort (*n* = 111) are listed in Table S1. Of the 206 patients in the training cohort, the median age [interquartile range] was 62 [56–68] years, and 168 (81.6%) patients were male. Forty-four (21.4%) patients experienced CIP during follow-up. The median follow-up time was 419 days [20-1369] days, and the median time to develop CIP was 105 days [20–183] days. Compared with the training cohort, more patients in the validation cohort received first-line ICI treatment (*P* = 0.001). No statistically significant differences in other variables were found between the training and validation cohorts.

Demographic characteristics of patients who did and did not experience CIP are shown in Table 1. In the CIP group, the patients were older (*P* = 0.025). In addition, there was a significant difference in the distribution of tumor histological types between CIP patients and non-CIP patients: although nonsquamous cell lung cancer was the main type of non-small cell lung cancer, squamous cell carcinoma accounted for a larger proportion in the CIP group (*P* = 0.049). The grading, radiological pattern and outcome of CIP are shown in Table S2.

**Table 1:** Baseline characteristics of training cohort patients between outcome groups

Variables	CIP			Grade 2–4 CIP			Grade 3–4 CIP		
	+(*n* = 44)	-(*n* = 162)	*P* value ^*^	+(*n* = 26)	-(*n* = 180)	*P* value ^*^	+(*n* = 16)	-(*n* = 190)	*P* value ^*^
Age, years ^a^	66 [62, 69]	61 [55, 67]	**0.025**	66 [62, 70]	61 [55, 67]	**0.002**	67 [62, 70]	61 [55, 67]	**0.018**
Gender ^b^									
Female	7 (15.9)	31 (19.1)	0.787	5 (19.2)	33 (18.3)	1	3 (18.8)	35 (18.4)	1
Male	37 (84.1)	131 (80.9)		21 (80.8)	147 (81.7)		13 (81.2)	155 (81.6)	
ECOG-PS ^b^									
0	15 (34.1)	69 (42.6)	0.127	8 (30.8)	76 (42.2)	0.538	5 (31.2)	79 (41.6)	0.524
1	21 (47.7)	80 (49.4)		15 (57.7)	86 (47.8)		10 (62.5)	91 (47.9)	
2	8 (18.2)	13 (8.0)		3 (11.5)	18 (10.0)		1 (6.2)	20 (10.5)	
Smoking history ^b^									
Never	8 (18.2)	47 (29.0)	0.212	6 (23.1)	49 (27.2)	0.834	4 (25.0)	51 (26.8)	1.000
Current/ex	36 (81.8)	115 (71.0)		20 (76.9)	131 (72.8)		12 (75.0)	139 (73.2)	
Clinical stage ^b^									
III	9 (20.5)	32 (19.8)	1.000	5 (19.2)	36 (20.0)	1.000	2 (12.5)	39 (20.5)	0.655
IV	35 (79.5)	130 (80.2)		21 (80.8)	144 (80.0)		14 (87.5)	151 (79.5)	
Histology ^b^									
Non-squamous	21 (47.7)	106 (65.4)	**0.049**	8 (30.8)	119 (66.1)	**0.001**	5 (31.2)	122 (64.2)	**0.019**
Squamous	23 (52.3)	56 (34.6)		18 (69.2)	61 (33.9)		11 (68.8)	68 (35.8)	
Prior thoracic radiation therapy ^b^									
No	30 (68.2)	130 (80.2)	0.134	20 (76.9)	140 (77.8)	1.000	14 (87.5)	146 (76.8)	0.502
Yes	14 (31.8)	32 (19.8)		6 (23.1)	40 (22.2)		2 (12.5)	44 (23.2)	
ICI drug target ^b^									
PD-L1	6 (13.6)	10 (6.2)	0.186	3 (11.5)	13 (7.2)	0.706	2 (12.5)	14 (7.4)	0.802
PD-1	38 (86.4)	152 (93.8)		23 (88.5)	167 (92.8)		14 (87.5)	176 (92.6)	
Treatment mode ^b^									
Combined therapy	28 (63.6)	111 (68.5)	0.666	17 (65.4)	122 (67.8)	0.984	11 (68.8)	128 (67.4)	1.000
Monotherapy	16 (36.4)	51 (31.5)		9 (34.6)	58 (32.2)		5 (31.2)	62 (32.6)	
Number of ICI cycles ^a^	12 [22]	8.00 [4, 14]	**0.013**	11 [5, 27]	9 [17]	0.258	8 [4, 15]	9 [4, 18]	0.55
Best tumor response ^b^									
PR	15 (34.1)	72 (44.4)	0.466	11 (42.3)	76 (42.2)	0.397	7 (43.8)	80 (42.1)	0.595
SD	23 (52.3)	72 (44.4)		10 (38.5)	85 (47.2)		6 (37.5)	89 (46.8)	
PD	6 (13.6)	18 (11.1)		5 (19.2)	19 (10.6)		3 (18.8)	21 (11.1)	
Line of ICI therapy ^b^									
1	15 (34.1)	67 (41.4)	0.055	11 (42.3)	71 (40.3)	0.152	5 (31.2)	77 (40.5)	0.331
2	22 (50.0)	62 (38.3)		12 (46.2)	72 (40.9)		10 (62.5)	74 (39.0)	
≥ 3	6 (13.6)	29 (17.9)		2 (7.7)	33(18.8)		1 (6.3)	34 (17.9)	
Missing data	1 (2.3)	4 (2.4)		1 (3.8)	4 (2.4)		0	5 (2.6)	
PD-L1 expression ^b^(*n* = 88)			0.793			0.730			0.422
< 1%	11 (57.9)	43 (62.3)		11 (57.9)	43 (62.3)		3 (42.9)	51 (63.0)	
≥ 1%	8 (42.1)	26 (37.7)		4 (44.4)	30 (38.0)		4 (57.1)	30 (37.0)	
EGFR mutation or ALK fusion ^b^(*n* = 78)									
No	15 (93.8)	52 (83.9)	0.444	7 (87.5)	60 (85.7)	0.891	5 (83.3)	62 (86.1)	0.851
Yes	1 (6.3)	10 (16.1)		1 (12.5)	10 (14.3)		1 (16.7)	10 (13.9)	
ILA ^b †^									
Without ILA	23 (52.3)	114 (70.4)	0.081	14 (53.8)	123 (68.3)	0.173	10 (62.4)	127 (66.8)	0.926
Equivocal ILA	10 (22.7)	22 (13.6)		4 (15.4)	28 (15.6)		3 (18.8)	29 (15.3)	
With ILA	11 (25.0)	26 (16.0)		8 (30.8)	29 (16.1)		3 (18.8)	34 (17.9)	
Fibrosis percentage in:									
Whole lung ^c^	1.25 (3.43)	0.62 (1.62)	0.081	1.69 (4.41)	0.62 (1.55)	**0.016**	1.88 (5.55)	0.66 (1.55)	**0.028**
LUL ^c^	1.43 (5.58)	0.47 (1.53)	0.053	0.88 (1.55)	0.54 (1.76)	0.347	2.53 (9.13)	0.52 (1.52)	0.008
LLL ^c^	1.43 (3.85)	1.76 (7.22)	0.774	1.92 (7.17)	0.49 (1.52)	**0.020**	0.84 (3.01)	1.76 (6.85)	0.596
RUL ^c^	1.14 (2.72)	1.00 (2.58)	0.755	1.11 (2.90)	1.02 (2.57)	0.866	1.15 (3.39)	1.02 (2.54)	0.849
RML ^c^	0.14 (0.33)	0.20 (0.66)	0.551	0.12 (0.34)	0.20 (0.64)	0.561	0.00 (0.00)	0.20 (0.63)	0.197
RLL ^c^	1.02 (2.29)	0.79 (4.28)	0.731	1.30 (2.81)	0.77 (4.07)	0.527	0.68 (1.62)	0.85 (4.07)	0.863
Lobes involved Fibrosis ^b^									
0	14 (31.8)	59 (36.4)	**0.041**	10 (38.5)	63 (35.0)	0.127	9 (56.2)	64 (33.7)	0.120
1	4 (9.1)	35 (21.6)		2 (7.7)	37 (20.6)		1 (6.2)	38 (20.0)	
2	13 (29.5)	22 (13.6)		8 (30.8)	27 (15.0)		4 (25.0)	31 (16.3)	
≥ 3	13 (29.5)	46 (28.4)		6 (23.1)	53 (29.4)		2 (12.5)	57 (30.0)	
GGO percentage in:									
Whole lung ^c^	1.08 (3.38)	0.17 (0.68)	**0.002**	1.70 (4.31)	0.18 (0.65)	**< 0.001**	1.63 (3.83)	0.26 (1.35)	**0.002**
LUL ^c^	1.13 (6.19)	0.17 (1.62)	0.077	1.19 (3.50)	0.21 (0.92)	**0.021**	0.24 (0.90)	0.39 (3.32)	0.865
LLL ^c^	0.06 (0.20)	0.13 (0.97)	0.650	1.73 (8.00)	0.18 (1.57)	0.572	0.00 (0.00)	0.13 (0.90)	0.580
RUL ^c^	0.34 (1.20)	0.13 (0.72)	0.137	0.27 (1.32)	0.16 (0.76)	0.539	0.44 (1.68)	0.15 (0.74)	0.193
RML ^c^	0.15 (0.71)	0.63 (4.79)	0.504	0.25 (0.92)	0.57 (4.55)	0.720	0.40 (1.16)	0.54 (4.42)	0.903
RLL ^c^	1.99 (8.09)	0.20 (1.90)	**0.010**	3.25 (10.41)	0.20 (1.81)	**< 0.001**	5.27 (13.01)	0.19 (1.76)	**< 0.001**
Lobes involved GGO ^b^									
0	23 (52.3)	114 (70.4)	**0.048**	14 (53.8)	123 (68.3)	0.123	11 (68.8)	126 (66.3)	0.831
1	14 (31.8)	31 (19.1)		8 (30.8)	37 (20.6)		4 (25.0)	41 (21.6)	
2	6 (13.6)	9 (5.6)		4 (15.4)	11 (6.1)		1 (6.2)	14 (7.4)	
≥ 3	1 (2.3)	8 (4.9)		0 (0.0)	9 (5.0)		0 (0.0)	9 (4.7)	
Consolidation percentage in:									
Whole lung ^c^	2.40 (3.10)	2.05 (3.50)	0.557	2.27 (3.30)	2.11 (3.44)	0.821	2.04 (2.86)	2.13 (3.46)	0.913
LUL ^c^	2.05 (4.95)	3.28 (9.86)	0.426	2.67 (6.92)	2.57 (5.21)	0.933	2.02 (5.03)	3.10 (9.31)	0.649
LLL ^c^	2.15 (6.00)	1.66 (5.56)	0.615	2.53 (5.74)	3.09 (9.44)	0.769	1.41 (4.23)	1.80 (5.75)	0.795
RUL ^c^	4.48 (10.63)	3.66 (9.68)	0.627	3.06 (8.60)	3.95 (10.05)	0.668	2.31 (6.02)	3.96 (10.12)	0.521
RML ^c^	2.16 (5.85)	1.37 (5.32)	0.390	2.68 (7.08)	1.37 (5.16)	0.251	2.45 (5.62)	1.46 (5.42)	0.489
RLL ^c^	3.79 (9.37)	3.76 (10.17)	0.986	3.06 (7.25)	3.87 (10.33)	0.699	2.90 (4.74)	3.84 (10.30)	0.716
Lobes involved consolidation ^b^									
0	6 (13.6)	19 (11.7)	0.667	5 (19.2)	20 (11.1)	0.547	3 (18.8)	22 (11.6)	0.644
1	12 (27.3)	53 (32.7)		6 (23.1)	59 (32.8)		4 (25.0)	61 (32.1)	
2	13 (29.5)	35 (21.6)		7 (26.9)	41 (22.8)		5 (31.2)	43 (22.6)	
≥ 3	13 (29.5)	55 (34.0)		8 (30.8)	60 (33.3)		4 (25.0)	64 (33.7)	
WBC (10^9^/L) ^c^	6.95 (2.14)	6.89 (2.58)	0.902	6.81 (2.11)	6.92 (2.54)	0.847	6.87 (2.39)	6.91 (2.50)	0.960
NEU (10^9^/L) ^c^	4.65 (1.71)	5.72 (7.90)	0.392	4.48 (1.65)	5.64 (7.51)	0.446	4.79 (1.63)	5.55 (7.33)	0.680
LYM (10^9^/L) ^c^	1.50 (0.68)	1.81 (3.15)	0.526	1.55 (0.72)	1.77 (2.99)	0.704	1.38 (0.70)	1.78 (2.91)	0.585
PLT (10^9^/L) ^c^	249.90 (87.56)	246.19 (93.93)	0.821	241.92 (89.88)	247.82 (92.96)	0.767	259.62 (100.60)	245.82 (91.73)	0.569
MONO (10^9^/L) ^c^	0.62 (0.23)	0.57 (0.27)	0.390	0.62 (0.26)	0.58 (0.26)	0.483	0.59 (0.23)	0.58 (0.27)	0.909
AEC (10^9^/L) ^c^	0.15 (0.12)	0.19 (0.31)	0.440	0.14 (0.12)	0.19 (0.29)	0.441	0.15 (0.11)	0.19 (0.29)	0.574
Hb (g/L) ^c^	129.02 (18.65)	128.97 (20.59)	0.987	127.56 (20.38)	129.20 (20.14)	0.706	127.50 (24.58)	129.12 (19.73)	0.759
ALB (g/L) ^c^	41.00 (4.58)	40.77 (4.81)	0.786	41.19 (4.88)	40.76 (4.74)	0.671	41.95 (4.55)	40.72 (4.77)	0.320

^a^ Values are given in median [interquartile range]

^b^ Values are given in number (%)

^c^ Values are given in mean (standard deviation)

^*^ Significant *P* values < 0.05 are in bold

^†^ ILA according to the radiologists

Table 1 also summarize the pretreatment CT findings. Among the 206 patients in the training cohort, the radiologists identified 137 (66.5%) patients without ILA, 32 (15.5%) patients with equivocal ILA, and 37 (18.0%) patients with ILA. According to the AI evaluation, there were 153 (74.3%) patients without ILA and 53 (25.7%) patients with ILA. The existence of ILA according to the AI evaluation correlated well with the existence of ILA according to the radiologists (contingency coefficient *r* = 0.77; *P*<0.001). The CT results before ICI treatment revealed that the mean percentage fibrosis extent was 0.75 ± 2.14, and the percentage of GGO extent according to the AI evaluation was 0.37 ± 1.70. The percentage of consolidation volume according to the AI evaluation was 2.13 ± 3.41. As shown in Table 1, CT quantitative assessment of lung lesions showed that the lung GGO extent of patients with CIP was higher than that of patients without CIP (*P* = 0.002), and pretreatment lung GGOs in patients with CIP occupied more lobes (*P* = 0.048).

### Model development

For the variables “HGB”, “PLT”, “WBC”, “NEUT”, “LYM”, “EOS”, and “MONO”, data were missing for 22 out of 206 patients (10.68%), “ALB” data were missing for 24 out of 206 patients (11.17%), and “LDH” data were missing for 39 out of 206 patients (18.93%). In the variable “number of ICI cycles”, data were missing for 5 out of 206 patients (2.43%), and in the variable “line of ICI therapy”, data was missing for 19 patients (9.22%). The main reasons for missing values were lost to follow-up or omissions of follow-up information. PD-L1 expression information from medical records was available in 88 patients, and EGFR/ALK mutation status was available in 78 cases in the training set. Baseline C-reactive protein (CRP) levels were available for only in 35 patients in the training set. The data for all other variables were complete. Missing values were imputed using random forest imputations, and the missing values of PD-L1 expression, EGFR/ALK gene mutation status and CRP had not been imputed and were not included in the development of the model. After multiple imputation, the data of all 206 patients were complete and could be used to develop a prediction model.

Forty-three variables (shown in Table 1) were included in the LASSO regression. After LASSO regression selection (Fig. S1), no variable remained a significant predictor of CIP. Seven variables remained significant predictors of grade ≥ 2 CIP, including histology, age, GGO percentage in whole lung, fibrosis percentage in whole lung, GGO percentage in the right lower lung, the number of involved lung lobes involved in GGOs and number of ICI cycles. Three variables remained significant predictors of high-grade CIP, including age, histology, and GGO percentage in the right lower lung.

Multivariate logistic regression analysis using backward stepwise model selection showed that the AI-evaluated percentage GGO extent was an independent predictor of the presence of grade ≥ 2 CIP (odds ratio (OR), 1.446; 95% confidence interval (CI): 1.103–2.257, *P* = 0.045) after adjustment for age and histology. Preexisting GGO extent in the right lower lung (OR, 1.157; 95% CI: 1.055–1.341, *P* = 0.009) and histology (OR, 4.734; 95% CI: 1.502–18.379, *P* = 0.012) were independent predictors of the existence of high-grade CIP. Compared with patients without high-grade CIP, patients with high-grade CIP were more likely to have a larger GGO extent in the right lower lung before ICI treatment. Fig. 2 shows typical cases. To enhance the clinical interpretability of the results, we evaluated the GGO extent as both a continuous and categorical variable, as shown in Table S3. As the findings from the analyses based on binary and continuous variables were consistent and more readily interpretable, binary GGO extent was employed in constructing both Model 1 and Model 2 in subsequent analyses. The optimal cutoff value for GGO extent was determined to be 1.01% after adjusting for histology and age, while the optimal cutoff value for preexisting GGO extent in the right lower lung was found to be 2.55% after adjusting for histology.

**Fig. 3:**
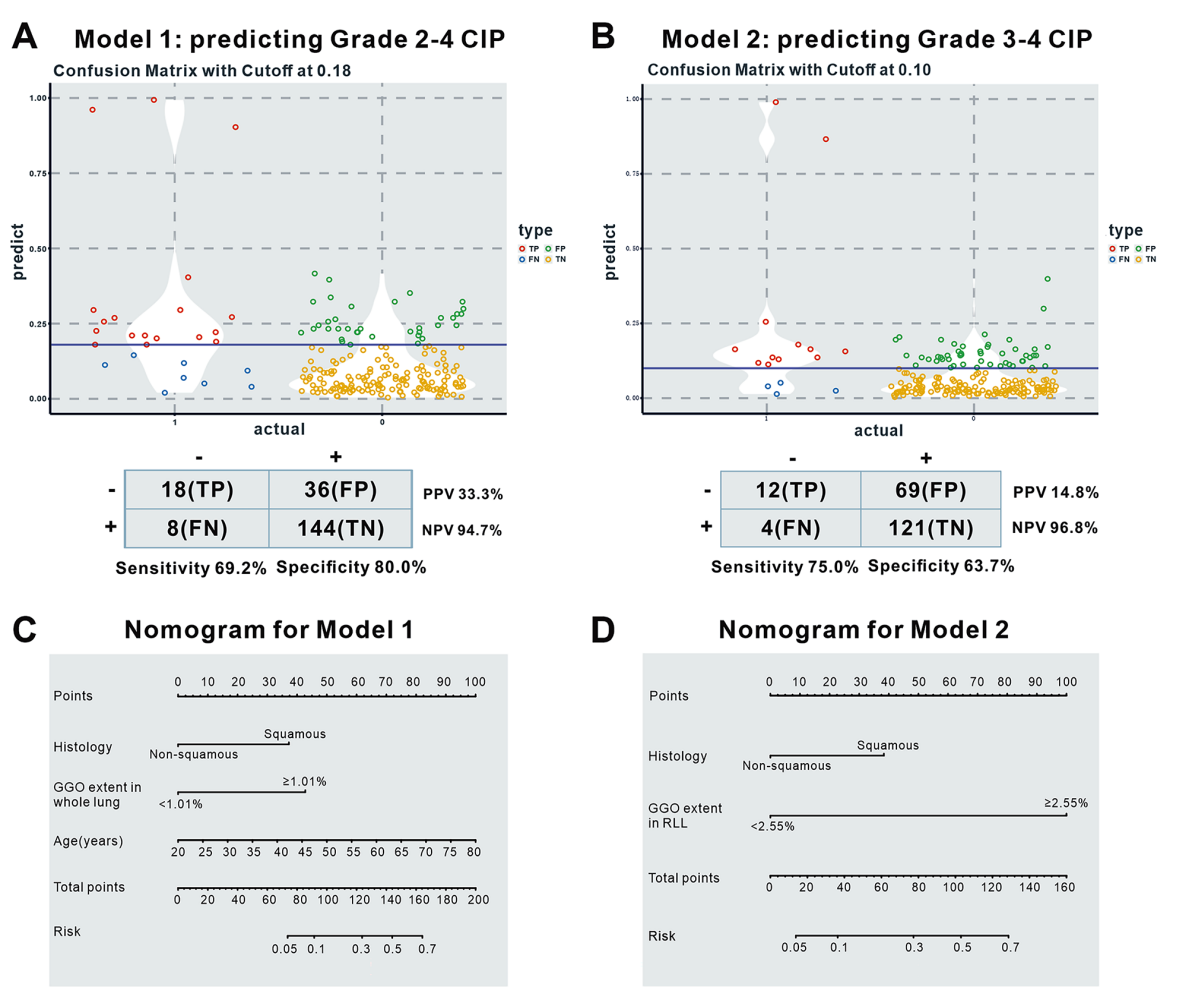
Confusion matrix plots (**A**) Model 1 and (**B**) Model 2. Classification tables showing the actual and predicted number of patients with or without different grades of CIP and their corresponding sensitivity, specificity, PPV and NPV, using (**A**) Model 1 (probability threshold 0.18) and (**B**) Model 2 (probability threshold 0.10). Nomograms of (**C**) Model 1 and (**D**) Model 2. Abbreviations: CIP: checkpoint inhibitor pneumonitis, TP: true positive, FP: false positive, FN: false negative, TN: true negative, PPV: positive predictive value, NPV: negative predictive value. GGO: ground-glass opacity; RLL: right lower lobe

Table S3 shows the models that can be used to compute the probability of grade ≥ 2 CIP (Model 1) and high-grade CIP (Model 2). Fig. 3A, B presents the probability cutoff values for Model 1 and Model 2, along with their corresponding sensitivity, specificity, PPV, and NPV, as well as the confusion matrix plots, with probability thresholds of 0.18 and 0.10 chosen for Model 1 and Model 2, respectively. Nomograms based on Model 1 and Model 2 were developed to allow clinicians to calculate the risk that a patient with ICI treatment will develop CIP requiring clinical management by knowing the values of the variables in the models (Fig. 3C, D).

### Performance of the models

**Fig. 4:**
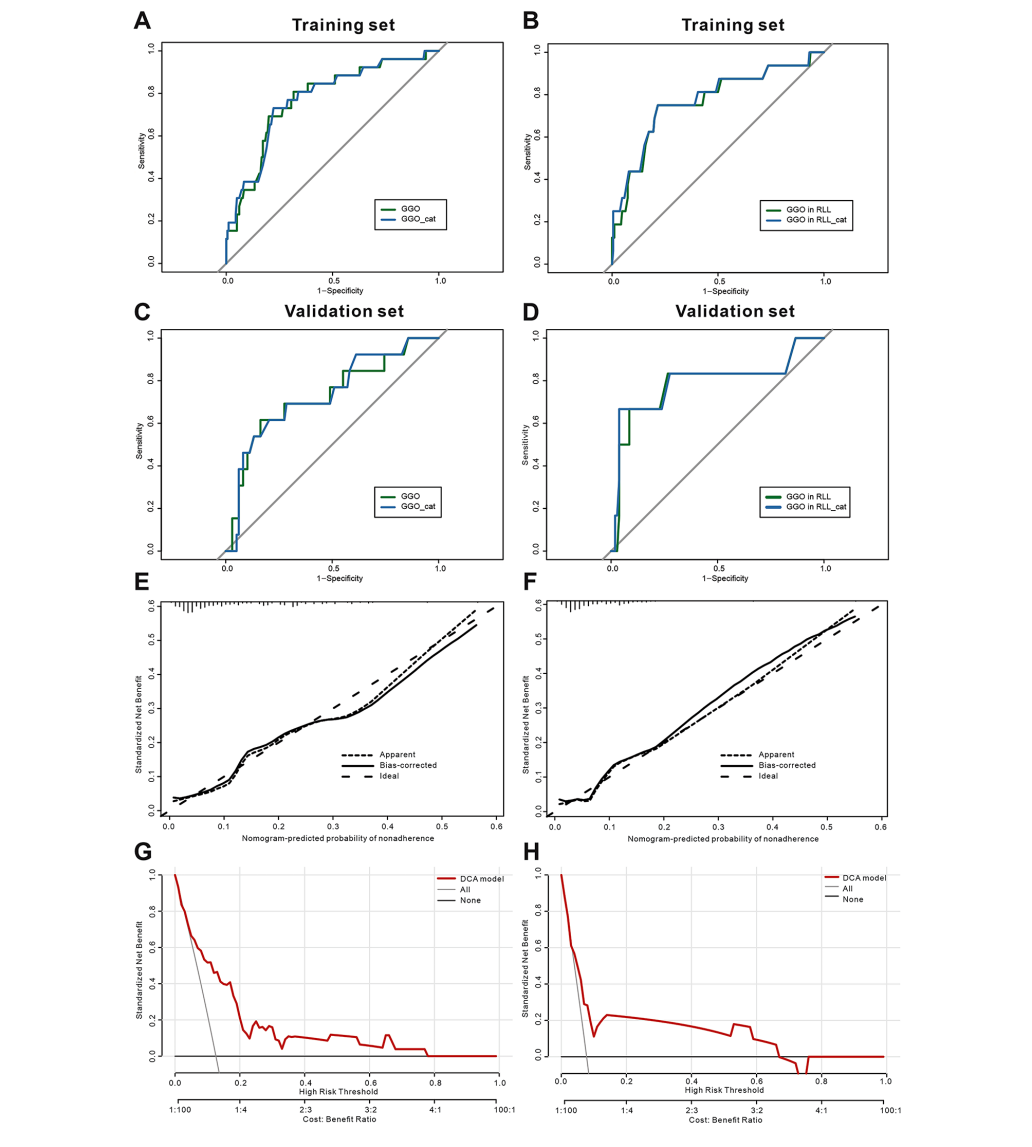
Receiver operating characteristic curve of (**A**) Model 1 and (**B**) Model 2 in training cohort, and (**C**) Model 1 and (**D**) Model 2 in validation cohort. Calibration plots with the observed probability of grade ≥ 2 CIP by predicted probability of (**E**) Model 1, and calibration plots with the observed probability of grade ≥ 3 CIP by predicted probability of (**F**) Model 2. The DCA curve of (**G**) Model 1 and (**H**) Model 2 in training cohort. Abbreviations: GGO: ground-glass opacity; GGO_cat: extent of ground-glass opacity in categorical variable; RLL: right lower lobe

The AUCs of Model 1 and Model 2 were 0.775 (95% CI: 0.692-0.800) and 0.735 (95% CI: 0.637–0.750), respectively, indicating quite good discriminatory ability. Fig. 4A, B shows the receiver operating characteristic curves of both models. By internal bootstrap validation, the mean AUC based on data from the training cohort was 0.783 for Model 1 and 0.731 for Model 2. Both H-L goodness-of-fit tests were nonsignificant (chi-squared = 10.411, *P* = 0.237 and chi-squared = 4.316, *P* = 0.828, Table S4). Both models showed good calibration, as indicated by calibration plots showing good agreement between the actual and predicted probabilities (Fig. 4E, F). DCA plots for the logistic regression models predicting the risk of CIP showed that the models had clinical utility in guiding treatment decisions across a range of threshold probabilities (Fig. 4G, H).

The validation cohort included 111 patients, 20 (18.0%) of whom developed CIP, 6 (5.4%) of whom developed grade ≥ 3 CIP. The AUCs of Model 1 and Model 2 for patients in the validation cohort were 0.732 (95% CI, 0.615–0.796) and 0.795 (95% CI, 0.667–0.962), respectively (Fig. 4C, D). The accuracy of Model 1 and Model 2 in the validation cohort was similar to that in the training cohort.

### Exploration of baseline CT findings in predicting the efficacy of ICI therapy

**Fig. 5:**
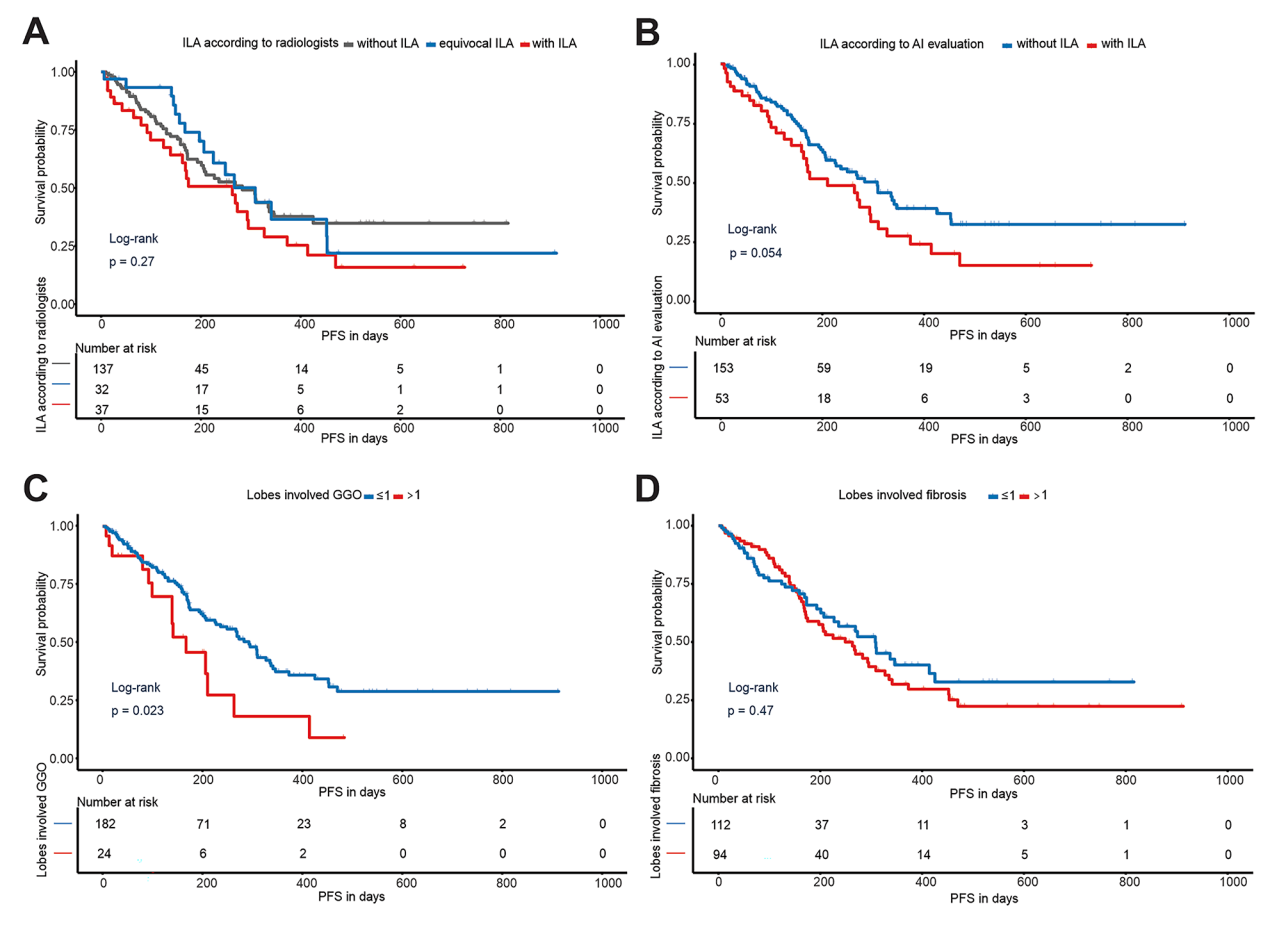
Kaplan-Meier curves of progression-free survival. (**A**) Comparison between three groups on the basis of ILA according to the radiologists; (**B**) Comparison between two groups on the basis of ILA according to the AI evaluation; (**C**) Comparison between two groups on the basis of lobes involved GGO; (**D**) Comparison between two groups on the basis of the number of involved lung lobes. Abbreviations: ILA: interstitial lung abnormalities; AI: artificial intelligence; GGO: ground-glass opacity

Fig. 5 shows Kaplan-Meier curves comparing PFS between groups. The existence of ILA according to radiologists was not a predictor of PFS (*P* = 0.270, Fig. 5A), whereas patients with preexisting ILA according to AI evaluation showed a trend toward poor PFS (*P* = 0.054, Fig. 5B). GGOs (*P* = 0.023, Fig. 5C) but not fibrosis lesions (*P* = 0.470, Fig. 5D) involving more than one lobe at baseline CT were a risk factor for PFS. Univariable and multivariable Cox regression analysis results for each factor to predict PFS are shown in Table S5: neither fibrosis extent nor GGO extent could predict PFS. After adjusting for ICI drug and peripheral blood lymphocyte count, GGOs involving more than one lobe remained associated with poorer PFS (hazard ratio (HR), 2.098; 95% CI: 1.178–3.725; *P* = 0.012).

## Discussion

In this study, we developed and validated two prediction models that can be used to predict the probability of CIP during ICI therapy for patients with advanced non-small cell lung cancer. The first model predicted a patient’s probability of grade ≥ 2 CIP and held 3 predictors constant, including GGO percentage in whole lung, age and histology. The second model predicted the probability of grade ≥ 3 CIP and had 2 predictors, including GGO percentage in the right lower lung and histology. Both models showed good discriminative ability and accurate prediction, with Model 1 performing better.

To our knowledge, this is the first study to quantitatively report the baseline CT findings, including the ILA component, extent and distribution, as risk factors for CIP. We found that determining the quantitative GGO extent with a deep learning algorithm was useful for evaluating the existence of CIP, especially symptomatic CIP at pretreatment CT, and for risk stratification of patients with ICI-treated NSCLC. Our study sought to examine the potential of identifying GGO regions on baseline chest CT images as a means of predicting the development of CIP, and to evaluate the utility of quantitative analysis for classifying these regions as high-risk or low-risk. The optimal cutoff values identified for GGO extent in the whole lung (1.01%) and preexisting GGO extent in the right lower lung (2.55%) might inform clinical decision-making and management strategies for patients. And the predictive models could be readily applied in clinical settings, whether via similar computer-aided detection software or rough assessments conducted by radiologists or oncologists. This holds notable clinical significance, as it enables the estimation of CIP risk in a more accessible and streamlined manner.

Previous studies have shown that ILA in cancer patients increases the risk of developing severe pneumonitis as a side effect of systemic chemotherapy, radiation therapy and immune checkpoint inhibitors [25, 32–34]. One of the challenges in developing a suitable prediction model of CIP was the lack of objectivity in ILA evaluation. First, visually evaluating the extent of ILAs may not be an easy task for radiologists. Second, the distinction between fibrotic and nonfibrotic ILAs is challenging. In the field of medical imaging, advances in the field of artificial intelligence and machine learning seem to offer incredible opportunities [35, 36]. Quantitative imaging techniques have been successfully used to evaluate ILAs. Iwasawa et al. [37], for instance, observed that the volume ratio of fibrosis at preoperative CT, as measured by using a computer-aided quantification system, was an independent predictor of lower disease-free survival in patients with lung cancer. Our study, which included AI labeling, quantitative imaging and computational analysis, might improve the objectiveness and reproducibility and might be efficiently employed in a prediction model of CIP. It is worth mentioning that discrepancies between visual and automated assessment in GGOs and fibrotic opacities have been described [38]; thus, in this study, we used a multitask deep learning algorithm combined with manual inspection to overcome this limitation. In addition, risk factors for developing CIP in previous studies, including older age [14] and squamous histology [11], were verified in the present study.

In addition, as mentioned above, multivariable regression analysis showed that CT quantitative assessment of baseline lung GGO but not fibrosis percentage was an independent risk factor for grade ≥ 2 CIP. These results are consistent with those of previous studies [25, 39, 40]. Mechanically, GGOs may represent potentially reversible inflammation and the infiltration of lymphocytes, while the reticular structure and the honeycomb structure tend to reflect architectural distortion of the alveola, reflecting fibrosis rather than inflammation. Several studies have suggested that T cells are activated and infiltrate the lung tissue of CIP patients [41–43]. The development of CIP may also be attributed to the excessive activation of the immune system induced by inflammatory cytokines, which leads to off-target lung destruction by cytotoxic T cells [44–46]. These findings may explain why the presence of GGOs is a more important risk factor for CIP in this study and may also explain the conclusions in previous literature that no association exists between usual interstitial pneumonia (UIP) patterns and pneumonitis risk [23, 47].

Moreover, we also found that patients with scattered GGOs in multiple lobes before treatment tended to have poor PFS after immunotherapy. Worse outcomes have been reported in patients with advanced lung cancer with ILA [32]. ILA also influences disease-free survival in patients with resectable NSCLC [37]. However, the quantitative evaluation of GGOs in patients with lung cancer and their influence on the efficacy of ICI therapy have not yet been fully investigated. This study provided a preliminary clue to the exploration of this question. Our study may lead to the development of a noninvasive method for capturing the status of the baseline inflammatory/immune microenvironment of the lungs, which may assist in the development of efficacy prediction models for immunotherapy in advanced NSCLC patients. Our results further reflected the importance of pretreatment CT assessment before ICI intervention.

Our research had several limitations. Firstly, it was a retrospective study conducted in a single center, resulting in a moderate level of evidence. Additionally, despite internal validation of the predictive models, external validation in independent cohorts has not been performed. These constraints posed challenges to the generalizability of our findings. We will conduct future prospective studies, with a specific focus on externally validating the robustness and applicability of proposed models across multiple medical centers and independent cohorts. This will contribute to a comprehensive evaluation of our models, potentially laying a foundation for their future implementation in clinical practice. Secondly, the wide CI of the OR for the variable “GGO percentage in whole lung” in Model 2 highlights the importance of considering the potential sources of variability and uncertainty in our findings. The effect of this variable may be influenced by other factors that were not included in our analysis. Thus, caution should be exercised when interpreting and applying the results of our study. Thirdly, the number of patients with CIP was relatively small, so the generalization of the results was limited. Furthermore, we used CT images obtained from a single center and software platform, which limited the universality of these results. However, the multitask deep learning algorithm is commercialized and has been reported to work effectively on different CT scanner manufacturers in many previous studies [48–50]. Further prospective multicenter validation studies with larger sample sizes are therefore necessary to solve these points.

## Conclusions

In summary, we developed and validated predictive models for symptomatic and severe CIP using a deep learning algorithm that accurately detected and quantified GGOs in CT images. Our findings highlight the importance of monitoring patients with multilobe-involved GGOs prior to ICI therapy, as they have a higher risk of CIP and poorer outcomes. The resulting models have notable clinical significance and can be easily applied in clinical settings. Further research can explore generalizability, improve sensitivity and specificity, and incorporate additional data for more personalized medicine.

### Electronic supplementary material

Below is the link to the electronic supplementary material.

Supplementary Material 1

## Data Availability

Associated codes used for data processing and analysis are publicly available from the GitHub using the following web link: https://github.com/wangxinyue1027/favorite-song.git. Data are accessible with reasonable request. All related data are included in the article and in additional online files or are available from the corresponding author upon reasonable request.

## Abbreviations

CIP: Checkpoint inhibitor pneumonitis
ECOG-PS: The Eastern Cooperative Oncology Group performance status
ICI: Immune checkpoint inhibitor
PD-L1: Programmed cell death protein ligand-1
PD-1: Programmed cell death protein-1
PR: Partial response
SD: Stable disease
PD: Progressive disease
EGFR: Epidermal growth factor receptor gene
ALK: Anaplastic lymphoma kinase gene
ILA: Interstitial lung abnormality
RUL: Right upper lobe
RML: Right middle lobe
RLL: Right lower lobe
LUL: Left upper lobe
LLL: Left lower lobe
GGO: Ground-glass opacity
WBC: White blood cell count
NEU: Neutrophil count
LYM: Lymphocyte count
PLT: Platelet count
MONO: Monocytes count
AEC: Absolute eosinophil count
Hb: Hemoglobin
ALB: Albumin
LDH: Lactate dehydrogenase
