# Supplementary material for: Quantification of preexisting lung ground glass opacities on CT for predicting checkpoint inhibitor pneumonitis in advanced non-small cell lung cancer patients
Source: BMC Cancer. 2024 Feb 26;24:269. doi: 10.1186/s12885-024-12008-z (PMC10895810; doi:10.1186/s12885-024-12008-z)
Supplement: Supplementary file 1 — Supplementary Material 1 [file 12885_2024_12008_MOESM1_ESM.docx]

# Supplementary Methods

***CT imaging evaluation***

Analysis of pretreatment CT images by using the dedicated multitask deep learning algorithm developed for pulmonary pneumonia (Beijing Deepwise & League of PhD Technology Co. Ltd, China). In this study, a dedicated multitask deep learning algorithm was developed for pulmonary pneumonia, which enabled simultaneous segmentation of pulmonary segments and lobes. A 3D U-Net was introduced for pixel-level classification with large-scale annotated data. And weak labels were annotated by drawing a box within each pulmonary segment to provide sufficient training data, minimizing the need for precise annotations. This allowed for training on a large amount of weakly labeled data in a short period, and an active learning approach was adopted to select the most informative samples for annotation and training, significantly improving training efficiency.

For pulmonary opacity detection and segmentation, a feature pyramid network was used as the backbone network to achieve excellent performance in detecting different symptoms of various scales. Images with different window widths and center values were input during training to provide more information. A multi-model fusion strategy was developed to integrate multiple diagnostic results from different models, further improving the final detection performance.

In order to ensure the accuracy of lung lobe and lesion segmentation, three methods were utilized. As reported by Wang et al. at the IEEE International Symposium on Biomedical Imaging in 2020, the proposed method achieved a dice score of 0.97 for lung lobe segmentation in the easy HOUSE dataset and 0.89 in the challenging LOLA dataset, demonstrating its effectiveness[1]. The common lobe Dice loss and boundary Dice loss were applied to detect lobar boundaries and enhance boundary recognition. Additionally, the model was trained using annotations labeled by experienced radiologists to accurately identify lesions, with the boundary Dice loss used as well. The segmentation results of the cases in the study were reviewed by a chest imaging expert with 15 years of experience (J.Z).

***Data analysis***

**Data quality and missing data**

Imputation for missing variables was considered if missing values were less than 20%. We used the R package “MICE” to impute missing data.

Variable selection and model development

Potential predictors were preselected based on expert opinion and a literature review[2-13]. The following variables were selected: age, gender, ECOG-PS, smoking history, clinical stage, histology, prior thoracic radiation therapy, ICI drug, treatment mode, number of ICI cycles, best tumor response for ICI, line of ICI therapy, CT findings and laboratory findings. Forty-three variables were entered into the selection process. Least absolute shrinkage and selection operator (LASSO) regression was used to minimize potential collinearity and overfitting of the variables. The most predictive covariates were selected by the minimum λ that gives the minimum mean cross-validated error. The R package “glmnet” was used to perform LASSO regression.

Subsequently, variables identified by LASSO regression analysis were entered into logistic regression models. To further reduce the number of predictors in the multivariable logistic regression models, as the prevalence of outcome was relatively low, backward stepwise elimination based on the Wald test was used, and a liberal P value of 0.10 was used. For Model 1, three out of seven variables were introduced in a multivariable logistic regression model. For Model 2, only two predictors could be introduced. Optimal cut-off values were defined by the “CatPredi” R package for significant continuous variables. The variables that were consistently statistically significant were used to construct the model, which was then used to construct a nomogram.

**Performance of the model**

The performance of both models was assessed using Nagelkerke’s R^2^ and the Brier score. The ability of the models to discriminate between patients who experienced the outcomes and individuals who remained event-free was quantified as the area under the receiver operating characteristic curve (AUC). Additionally, sensitivity, specificity, positive predictive value (PPV), and negative predictive value (NPV) were calculated for both models to select probability cutoff values. The probability threshold was used to classify patients who were at high risk for the outcomes, and patients were considered at high risk if their predicted probabilities were at or above this threshold. A calibration plot was used to assess the agreement between the predicted probabilities and observed frequencies of the outcomes and a Hosmer and Lemeshow (H-L) goodness of fit test was calculated, with nonsignificant H-L statistics indicating good model fit. Furthermore, decision curve analysis (DCA) was performed to assess the clinical usefulness of the models.

**Validation**

For internal validation of the accuracy estimates and to reduce overfit bias, we used 400 bootstrap resamples. We also used data from our hospitals that were not included in the training cohort for further validation. The variables required for calculating the models from the validation cohort were collected, and AUCs were calculated to validate the accuracy of both models.

***Statistical analysis***

Continuous variables are described as the mean (standard deviation) or median [interquartile range], and categorical variables are described as numbers (percentages). Baseline characteristics between the training cohort and the test cohort, as well as variables between outcome groups in univariate analysis, were compared using Student's t test, Wilcoxon rank sum test, chi-square test or Fisher's exact test as appropriate. We also performed a chi-square test and calculated the contingency coefficient to analyze the correlation between the ILA determined by the radiologists and the AI-evaluated results. To examine the significance of clinical characteristics, laboratory findings and CT findings as predictors of PFS, univariate and multivariate Cox proportional hazard models were used. The Kaplan‒Meier method was employed to construct cumulative survival curves. Statistical analysis was performed with R software (version 4.2.1, R Foundation, Vienna, Austria), and a value of *P* < 0.05 was considered statistically significant.

# Supplementary Figure and Figure Legend


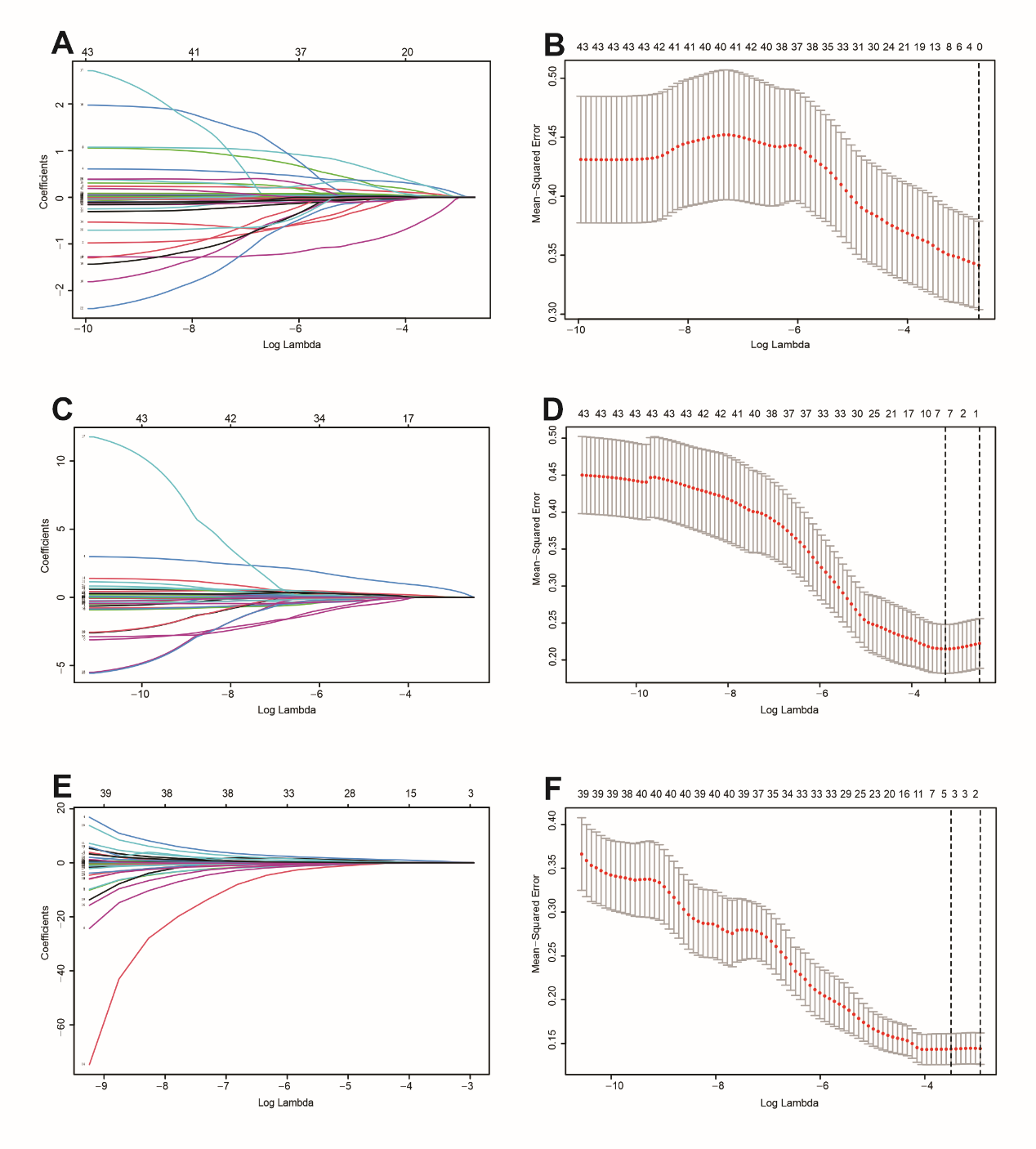


**Fig. S1** LASSO regression selection. Feature selection for predictors of CIP (A, B), grade≥2 CIP (C, D) and grade≥3 CIP (E, F) using the least absolute shrinkage and selection operator (LASSO) regression model. (A, C, E) LASSO coefficient profiles of the baseline features. (B, D, F) Tuning parameter (λ) selection in the LASSO model used 10-fold cross-validation via minimum criteria.

# Supplementary Tables

**Table S1** Baseline characteristics of patients in training cohort and validation cohort.

| Variables | Training cohort (N = 206) | Validation cohort (N = 111) | *P* value ^*^ |
| --- | --- | --- | --- |
| Age, years ^a^ | 62 [56, 68] | 61 [55, 67] | 0.175 |
| Gender ^b^ |  |  |  |
| Female | 38 (18.4) | 22 (19.8) | 0.883 |
| Male | 168 (81.6) | 89 (80.2) |  |
| ECOG-PS ^b^ |  |  |  |
| 0 | 84 (40.8) | 37 (33.3) | 0.317 |
| 1 | 101 (49.0) | 58 (52.3) |  |
| 2 | 21 (10.2) | 16 (14.4) |  |
| Smoking history ^b^ |  |  |  |
| Never | 55 (26.7) | 38 (34.2) | 0.202 |
| Current/ex | 151 (73.3) | 73 (65.8) |  |
| Clinical stage ^b^ |  |  |  |
| III | 41 (19.9) | 27 (24.3) | 0.440 |
| IV | 165 (80.1) | 84 (75.7) |  |
| Histology ^b^ |  |  |  |
| Non-squamous | 127 (61.7) | 73 (65.8) | 0.547 |
| Squamous | 79 (38.3) | 38 (34.2) |  |
| Prior thoracic radiation therapy ^b^ |  |  |  |
| No | 160 (77.7) | 79 (71.2) | 0.252 |
| Yes | 46 (22.3) | 32 (28.8) |  |
| ICI drug target ^b^ |  |  |  |
| PD-L1 | 16 (7.8) | 7 (6.3) | 0.802 |
| PD-1 | 190 (92.2) | 104 (93.7) |  |
| Treatment mode ^b^ |  |  |  |
| Combined therapy | 139 (67.5) | 63 (56.8) | 0.077 |
| Monotherapy | 67 (32.5) | 48 (43.2) |  |
| Number of ICI cycles ^a^ | 9 [4, 18] | 9 [4, 19] | 0.941 |
| Best tumor response ^b^ |  |  |  |
| PR | 87 (42.2) | 43 (38.7) | 0.505 |
| SD | 95 (46.1) | 50 (45.1) |  |
| PD | 24 (11.7) | 18 (16.2) |  |
| Line of ICI therapy ^b^ |  |  |  |
| 1 | 82 (39.8) | 59 (53.2) | **0.001** |
| 2 | 84 (40.8) | 23 (20.7) |  |
| ≥3 | 35 (17.0) | 29 (26.1) |  |
| Missing data | 5 (2.4) | 0 |  |
| PD-L1 expression ^b^ |  |  |  |
| <1% | 54 (26.2) | 30 (27.0) | 0.576 |
| ≥1% | 34 (16.5) | 23 (20.7) |  |
| Missing data | 118 (57.3) | 58 (52.3) |  |
| EGFR mutation or ALK fusion ^b^ |  |  |  |
| No | 67 (32.5) | 31 (27.9) | 0.328 |
| Yes | 11 (5.3) | 3 (2.7) |  |
| Missing data | 128 (62.2) | 77 (69.4) |  |
| ILA ^b †^ |  |  |  |
| Without ILA | 137 (66.5) | 69(62.2) | 0.693 |
| Equivocal ILA | 32 (15.5) | 18 (16.2) |  |
| With ILA | 37 (18.0) | 24 (21.6) |  |
| Fibrosis percentage in: |  |  |  |
| Whole lung ^c^ | 0.75 (2.14) | 0.53 (1.40) | 0.324 |
| LUL ^c^ | 0.67 (2.92) | 0.66 (2.18) | 0.275 |
| LLL ^c^ | 1.69 (6.64) | 0.36 (1.20) | 0.414 |
| RUL ^c^ | 0.19 (0.61) | 0.92 (2.95) | 0.733 |
| RML ^c^ | 0.67 (2.92) | 0.14 (0.74) | 0.570 |
| RLL ^c^ | 0.84 (3.93) | 0.89 (5.11) | 0.930 |
| Lobes involved Fibrosis ^b^ |  |  |  |
| 0 | 73 (35.4) | 38 (34.2) | 0.313 |
| 1 | 39 (18.9) | 30 (27.0) |  |
| 2 | 35 (17.0) | 19 (17.1) |  |
| ≥3 | 59 (28.6) | 24 (21.6) |  |
| GGO percentage in: |  |  |  |
| Whole lung ^c^ | 0.37 (1.70) | 0.41 (1.76) | 0.843 |
| LUL ^c^ | 0.38 (3.20) | 0.61 (4.32) | 0.583 |
| LLL ^c^ | 0.12 (0.87) | 0.43 (2.51) | 0.101 |
| RUL ^c^ | 0.18 (0.84) | 0.16 (0.84) | 0.916 |
| RML ^c^ | 0.53 (4.26) | 0.92 (5.77) | 0.491 |
| RLL ^c^ | 0.59 (4.14) | 0.13 (0.63) | 0.251 |
| Lobes involved GGO ^b^ |  |  |  |
| 0 | 137 (66.5) | 74 (66.7) | 0.272 |
| 1 | 45 (21.8) | 21 (18.9) |  |
| 2 | 15 (7.3) | 14 (12.6) |  |
| ≥3 | 9 (4.4) | 2 (1.8) |  |
| Consolidation percentage in: |  |  |  |
| Whole lung ^c^ | 2.13 (3.41) | 1.89 (3.70) | 0.572 |
| LUL ^c^ | 3.02 (9.04) | 3.03 (9.20) | 0.990 |
| LLL ^c^ | 1.77 (5.64) | 2.19 (8.62) | 0.597 |
| RUL ^c^ | 3.83 (9.87) | 2.72 (8.59) | 0.320 |
| RML ^c^ | 1.54 (5.43) | 1.15 (4.07) | 0.510 |
| RLL ^c^ | 3.77 (9.98) | 3.52 (9.67) | 0.829 |
| Lobes involved consolidation ^b^ |  |  |  |
| 0 | 25 (12.1) | 22 (19.8) | 0.055 |
| 1 | 65 (31.6) | 42 (37.8) |  |
| 2 | 48 (23.3) | 24 (21.6) |  |
| ≥3 | 68 (33.0) | 23 (20.7) |  |

^a^ Values are given in median [interquartile range]

^b^ Values are given in number (%)

^c^ Values are given in mean (standard deviation)

^*^ Significant *P* values < 0.05 are in bold.

^†^ ILA according to the radiologists

Abbreviations: ECOG-PS: the Eastern Cooperative Oncology Group performance status; ICI: Immune checkpoint inhibitor; PD-L1: programmed cell death protein ligand-1; PD-1: programmed cell death protein-1; PR: partial response; SD: stable disease; PD: progressive disease; EGFR: epidermal growth factor receptor gene; ALK: anaplastic lymphoma kinase gene; ILA: interstitial lung abnormality; RUL: right upper lobe; RML: right middle lobe; RLL: right lower lobe; LUL: left upper lobe; LLL: left lower lobe; GGO: ground-glass opacity.

**Table S2** CIP grade, pattern and outcome training cohort and validation cohort

|  | CIP in training cohort (N = 44) | CIP in validation cohort (N = 23) | *P* value |
| --- | --- | --- | --- |
| CIP radiological pattern ^a^ |  |  |  |
| COP | 12 (27.3) | 7 (35.0) | 0.697 |
| GGO | 9 (20.5) | 5 (25.0) |  |
| NSIP | 15 (34.1) | 5 (25.0) |  |
| HP | 6 (13.6) | 1 (5.0) |  |
| others | 2 (4.5) | 2 (10.0) |  |
| CIP grade ^a^ |  |  |  |
| 1 | 18 (40.9) | 7 (35.0) | 0.686 |
| 2 | 10 (22.7) | 7 (35.0) |  |
| 3 | 14 (31.8) | 6 (30.0) |  |
| 4 | 2 (4.6) | 0 (0) |  |
| CIP outcome ^a^ |  |  |  |
| Improved | 31 (70.5) | 13 (65.0) | 0.966 |
| Stable/unchanged | 8 (18.2) | 4 (20.0) |  |
| Worsened | 3 (6.8) | 2 (10.0) |  |
| Unknown | 2 (4.5) | 1 (5.0) |  |

^a^ Values are given in number (%)

Abbreviations: CIP: checkpoint inhibitor pneumonitis; COP: cryptogenic organizing pneumonia; GGO: ground-glass opacity; NSIP: nonspecific interstitial pneumonia; HP: hypersensitive pneumonitis.

**Table S3** Multivariable Logistic Regression Models for Predicting CIP

| Variable | OR (95% CI) | *P* value |
| --- | --- | --- |
| Model 1—CIP grade ≥2 |  |  |
| Age, years (continuous) | 1.072(1.012-1.144) | 0.025 |
| Histology (non-squamous/squamous) | 4.754(1.893-13.293) | 0.002 |
| GGO percentage in whole lung (categorical) | 5.998(1.640-21.916) | 0.006 |
| Model 1 —CIP grade ≥2 |  |  |
| Age, years (continuous) | 1.066(1.007-1.138) | 0.036 |
| Histology (non-squamous/squamous) | 4.341(1.726-12.060) | 0.003 |
| GGO percentage in whole lung (continuous) | 1.446(1.103-2.257) | 0.045 |
| Model 2—CIP grade ≥3 |  |  |
| Histology (non-squamous/squamous) | 4.734(1.502-18.379) | 0.034 |
| GGO percentage in the right lower lung (categorical) | 27.054 (4.478-223.910) | ＜0.001 |
| Model 2 —CIP grade ≥3 |  |  |
| Histology (non-squamous/squamous) | 4.734(1.502-18.379) | 0.012 |
| GGO percentage in the right lower lung (continuous) | 1.157(1.055-1.341) | 0.009 |

Abbreviations: CIP: checkpoint inhibitor pneumonitis; GGO: ground-glass opacity; OR: odds ratio; CI: confidence interval

**Table S4** Performance of the prediction models

| Performance | Model 1 | Model 2 |
| --- | --- | --- |
| Nagelkerke’s R^2^ | 0.226 | 0.187 |
| Brier score | 0.098 | 0.107 |
| AUC (95% CI) | 0.775 (0.692-0.800) | 0.735 (0.637-0.750) |
| H-L goodness-of-fit test *P*-value | 0.237 | 0.828 |

Abbreviations: AUC: area under the receiver operating characteristic curve; CI: confidence interval; H-L goodness-of-fit test: Hosmer-Lemeshow goodness-of-fit test.

**Table S5** Results of univariable and multivariable Cox regression analysis of progression-free survival

| Variable | Univariable regression | | | Multivariable regression | | |
| --- | --- | --- | --- | --- | --- | --- |
|  | HR | 95% CI | *P* value ^*^ | HR | 95% CI | *P* value |
| Age, years (continuous) | 1.004 | 0.984-1.025 | 0.681 |  |  |  |
| Gender (female/male) | 0.716 | 0.432-1.185 | 0.194 |  |  |  |
| ECOG-PS (0/1/2) | 0.896 | 0.451-1.782 | 0.755 |  |  |  |
| Smoking history (never/current or ex) | 1.177 | 0.745-1.861 | 0.485 |  |  |  |
| Clinical stage (III/IV) | 0.933 | 0.569-1.528 | 0.782 |  |  |  |
| Histology (non-squamous/squamous) | 0.874 | 0.577-1.323 | 0.524 |  |  |  |
| Prior thoracic radiation therapy (no/yes) | 0.995 | 0.617-1.603 | 0.983 |  |  |  |
| ICI drug target (PD-L1/PD-1) | 0.420 | 0.223-0.792 | **0.007** | 0.384 | 0.203-0.728 | **0.003** |
| Treatment mode  (combined therapy/monotherapy) | 1.207 | 0.793-1.837 | 0.380 |  |  |  |
| Fibrosis percentage (continuous) | 1.003 | 0.927-1.086 | 0.937 |  |  |  |
| Fibrosis involved more than one lobe (no/yes) | 1.160 | 0.774-1.739 | 0.471 |  |  |  |
| GGO percentage (continuous) | 1.023 | 0.924-1.134 | 0.659 |  |  |  |
| GGO involved more than one lobe (no/yes) | 1.913 | 1.080-3.386 | **0.026** | 2.098 | 1.178-3.725 | **0.012** |
| Consolidation percentage (continuous) | 0.995 | 0.931-1.063 | 0.872 |  |  |  |
| Consolidation involved more than one lobe (no/yes) | 1.031 | 0.679-1.566 | 0.886 |  |  |  |
| WBC (continuous) | 1.024 | 0.940-1.115 | 0.589 |  |  |  |
| NEU (continuous) | 1.016 | 0.972-1.063 | 0.475 |  |  |  |
| LYM (continuous) | 1.055 | 1.009-1.104 | **0.019** | 1.063 | 1.016-1.112 | **0.008** |
| PLT (continuous) | 1.001 | 0.999-1.004 | 0.285 |  |  |  |
| MONO (continuous) | 1.192 | 0.542-2.62 | 0.662 |  |  |  |
| AEC (continuous) | 0.924 | 0.348-2.453 | 0.874 |  |  |  |
| Hb (continuous) | 1.003 | 0.992-1.013 | 0.625 |  |  |  |
| ALB (continuous) | 0.985 | 0.946-1.026 | 0.467 |  |  |  |
| LDH (continuous) | 1.001 | 1.000-1.002 | 0.268 |  |  |  |

^*^ Significant *P* values < 0.05 are in bold.

Abbreviations: HR: hazard ratio; CI: confidence interval; ECOG-PS: the Eastern Cooperative Oncology Group performance status; ICI: Immune checkpoint inhibitor; PD-L1: programmed cell death protein ligand-1; PD-1: programmed cell death protein-1; PR: partial response; SD: stable disease; PD: progressive disease; GGO: ground-glass opacity; WBC: white blood cell count; NEU: neutrophil count; LYM: lymphocyte count; PLT: platelet count; MONO: monocytes count; AEC: absolute eosinophil count; Hb: Hemoglobin; ALB: albumin; LDH: lactate dehydrogenase.

**References**

1. Wang X, Zhang Q, Zhou Z, Liu F, Yu Y, Wang Y, Gao W: **Evaluating multi-class segmentation errors with anatomical priors**. In: *2020 IEEE 17th International Symposium on Biomedical Imaging (ISBI): 2020*: IEEE; 2020: 953-956.

2. Nishino M, Giobbie-Hurder A, Hatabu H, Ramaiya NH, Hodi FS: **Incidence of Programmed Cell Death 1 Inhibitor-Related Pneumonitis in Patients With Advanced Cancer: A Systematic Review and Meta-analysis**. *JAMA Oncol* 2016, **2**(12):1607-1616.

3. Khunger M, Rakshit S, Pasupuleti V, Hernandez AV, Mazzone P, Stevenson J, Pennell NA, Velcheti V: **Incidence of Pneumonitis With Use of Programmed Death 1 and Programmed Death-Ligand 1 Inhibitors in Non-Small Cell Lung Cancer: A Systematic Review and Meta-Analysis of Trials**. *Chest* 2017, **152**(2):271-281.

4. Suresh K, Voong KR, Shankar B, Forde PM, Ettinger DS, Marrone KA, Kelly RJ, Hann CL, Levy B, Feliciano JL *et al*: **Pneumonitis in Non-Small Cell Lung Cancer Patients Receiving Immune Checkpoint Immunotherapy: Incidence and Risk Factors**. *J Thorac Oncol* 2018, **13**(12):1930-1939.

5. Fukihara J, Sakamoto K, Koyama J, Ito T, Iwano S, Morise M, Ogawa M, Kondoh Y, Kimura T, Hashimoto N *et al*: **Prognostic Impact and Risk Factors of Immune-Related Pneumonitis in Patients With Non-Small-Cell Lung Cancer Who Received Programmed Death 1 Inhibitors**. *Clin Lung Cancer* 2019, **20**(6):442-450 e444.

6. Cho JY, Kim J, Lee JS, Kim YJ, Kim SH, Lee YJ, Cho YJ, Yoon HI, Lee JH, Lee CT *et al*: **Characteristics, incidence, and risk factors of immune checkpoint inhibitor-related pneumonitis in patients with non-small cell lung cancer**. *Lung Cancer* 2018, **125**:150-156.

7. Delaunay M, Cadranel J, Lusque A, Meyer N, Gounant V, Moro-Sibilot D, Michot JM, Raimbourg J, Girard N, Guisier F *et al*: **Immune-checkpoint inhibitors associated with interstitial lung disease in cancer patients**. *Eur Respir J* 2017, **50**(2).

8. Owen DH, Wei L, Bertino EM, Edd T, Villalona-Calero MA, He K, Shields PG, Carbone DP, Otterson GA: **Incidence, Risk Factors, and Effect on Survival of Immune-related Adverse Events in Patients With Non-Small-cell Lung Cancer**. *Clin Lung Cancer* 2018, **19**(6):e893-e900.

9. Naidoo J, Wang X, Woo KM, Iyriboz T, Halpenny D, Cunningham J, Chaft JE, Segal NH, Callahan MK, Lesokhin AM *et al*: **Pneumonitis in Patients Treated With Anti-Programmed Death-1/Programmed Death Ligand 1 Therapy**. *J Clin Oncol* 2017, **35**(7):709-717.

10. Yamaguchi T, Shimizu J, Hasegawa T, Horio Y, Inaba Y, Yatabe Y, Hida T: **Pre-existing pulmonary fibrosis is a risk factor for anti-PD-1-related pneumonitis in patients with non-small cell lung cancer: A retrospective analysis**. *Lung Cancer* 2018, **125**:212-217.

11. Suzuki Y, Karayama M, Uto T, Fujii M, Matsui T, Asada K, Kusagaya H, Kato M, Matsuda H, Matsuura S *et al*: **Assessment of Immune-Related Interstitial Lung Disease in Patients With NSCLC Treated with Immune Checkpoint Inhibitors: A Multicenter Prospective Study**. *J Thorac Oncol* 2020, **15**(8):1317-1327.

12. Shankar B, Zhang J, Naqash AR, Forde PM, Feliciano JL, Marrone KA, Ettinger DS, Hann CL, Brahmer JR, Ricciuti B *et al*: **Multisystem Immune-Related Adverse Events Associated With Immune Checkpoint Inhibitors for Treatment of Non-Small Cell Lung Cancer**. *JAMA Oncol* 2020, **6**(12):1952-1956.

13. Lin X, Deng H, Yang Y, Wu J, Qiu G, Li S, Xie X, Liu M, Xie Z, Qin Y *et al*: **Peripheral Blood Biomarkers for Early Diagnosis, Severity, and Prognosis of Checkpoint Inhibitor-Related Pneumonitis in Patients With Lung Cancer**. *Front Oncol* 2021, **11**:698832.
